# Transactions of the Chicago Society of Physicians and Surgeons

**Published:** 1875-02-01

**Authors:** R. E. Starkweather


### Society Reports.

### TRANSACTIONS OF THE CHICAGO SOCIETY OF PHYSICIANS AND SURGEONS.

### REGULAR MEETING, JANUARY 11, 1875.

*Reported by R. E. Starkweather, M.D.*

DR. JOHN BARTLETT, President, in the chair.

#### TUMOR OF DENTINE.

Dr. Andrews reported a case of unusual interest and rarity, and accompanied the same with an exhibition of a microscopical preparation of a portion of the material removed, furnished him by Dr. Curtis.

A writer in the recent edition of Holmes' Surgery, cites, and knows only of twelve cases of tumor of dentine, of which only six are of this kind. Dr. Andrews had heard of but one case in this city similar to his.

Dental tumors are of two sorts. The first is a simple deformity of the tooth, its structure not being changed.

The second sort is where the teeth depart from the normal structure altogether; generally, the molar teeth are the ones thus affected and deformed.

The patient, a lady of eighteen years of age, exhibited a swelling on the side of the jaw, where the wisdom tooth should have presented itself, very much as though a projecting bony tumor had been produced by a simple periostitis. A few months after having first seen the patient, the tumor or cyst began to suppurate, and it was at once incised. Upon probing, something like dead bone was felt, and the prominent point of the tumor had ulcerated through the gum. The patient having been anæsthetized, a longitudinal incision was made along the gum, and ineffectual attempts made to extract a supposed tooth. At length, by using a pair of curved gouge forceps, a fragment was broken off. In this manner, most of the then projecting portion of tumor was removed, and carbolated lotions ordered. Eight weeks later, there had been a slight loosening of the mass, and a little elevation thereof; and under anæsthesia, by using the tooth forceps, a rounded, tuberous body was removed from the cavity where a tooth should have been, which, on examination, proved to be composed of dentine. Its length was an inch and a quarter, and its thickness half an inch.

SARCOMA AND CARCINOMA.

Dr. Andrews made some remarks as to the clinical history of the sarcoma and carcinoma, their differential diagnosis, microscopical and physical, illustrating the same by reports of cases, and by microscopical specimens and drawings, prepared and furnished by Dr. Curtis, of carcinoma of the breast. He related three cases, microscopical specimens of which were prepared and displayed to the society by Prof. Curtis. The first was a sarcoma from a patient who had five such tumors, and recently died of erysipelas and peritonitis. Two of the tumors had been excised and returned, but there was no infection of the lymphatic glands. The second case was a carcinoma of the uterus, which Prof. Curtis diagnosed by the microscope during the life of the patient. The subsequent history and death confirmed his diagnosis. The third case was a carcinoma excised, by Prof. Andrews, from the side of the foot nearly two years ago. It has not yet returned.

Incidentally, Dr. Andrews remarked that it seemed to him that there were more cases of cancerous growths in northern Wisconsin, than in regions south of us; that consumption and cancer often prevailed in the same climates, and were more abundant the nearer one approaches to the sea coast of this country; and at equal distances from the sea, statistics show that they are more common at the north and less frequent as we go south.

HISTOLOGY OF CARCINOMA.

First, there is a fibrous frame-work in which are impacted a quantity of small, round cells, that is to say, bands of fibres interlace and leave lacunæ, and these are packed full of rounded cells. The best histologists consider these lacunæ to be lymphatic spaces, lined with the same endothelium as the lymphatic vessels, and constituting, in fact, a part of the lymphatic system. Hence the round cancer cells are contained in the interior of the lymphatic cavities, and, being easily detached from each other, whenever any of them become loose they float along the absorbent vessels until they are stopped at the first set of lymphatic glands. There they grow and produce another cancer. This is the reason why carcinoma regularly infects the absorbent glands. A characteristic of these cells in carcinoma is, that they can be easily brushed out, leaving the cavities or lacunæ empty—they can thus be easily conveyed through the body.

There is a certain class of tumors hard to destroy, called the sarcoma or flesh tumor. A few weeks ago, a woman, eighteen years of age, presented herself to me for treatment, upon whom there were several of these growths. They had been enlarging and increasing for upwards of two years. A year ago, two had been removed, but both returned, and one became larger than it had previously been. The tumors were from the size of a hazel nut to two inches in diameter — hard, not painful — the neighboring lymphatic glands were normal in every respect. There were two on the right thigh, but the inguinal glands were sound; there was one tumor in the left inguinal region of the abdomen, and suppurating. In every case, the lymphatic glands were perfectly normal, for sarcoma does not develop in the lymphatic spaces. Its cells are outside of them, hence are not taken into the absorbents. The cells of the sarcoma adhere more closely to each other than do those of carcinoma; they are spindle-shaped, and more like a fibroid tumor, so that it is sometimes hard to distinguish between sarcoma and fibroid tumors.

The sarcoma, unlike the carcinoma, has no frame-work apart from the cells. As a general rule, a diagnosis can be made between carcinoma and sarcoma. The hope of cure by excision, is greater in the latter than in the former.

Dr. Hyde thought it a difficult point as to whether a medullary sarcoma might not be considered cancerous. It was somewhat remarkable that in a recent discussion on cancer, in New York, Dr. Willard Parker had said that cancer was no longer to be considered as hereditary; that it often occurred in families with no previous history of cancer.

Dr. Curtis thought that there was a difference between sarcoma and carcinoma; the former throughout is made up of similar tissue—the latter is made up of entirely different elements. The colloid material is a degeneration of cancerous material, just as cancer may undergo fatty degeneration. The colloid is a transformation; it is an exaggerated species of cancer, the cells greatly dilated. In cancer, there is no shading off of the cells into the fibrous tissue. The term cancer, like that of consumption, has been abandoned by some authors —it is too general an expression.

After further discussion by Drs. Simon, Hyde, Merriman and Andrews, the society adjourned.

